# Operations Research to Solve Kidney Allocation Problems: A Systematic Review

**DOI:** 10.3390/healthcare11050768

**Published:** 2023-03-06

**Authors:** Nigar Sekercioglu, Rui Fu

**Affiliations:** 1 Telfer School of Management, University of Ottawa, Ottawa, ON K1N 6N5, Canada; 2 Department of Health Research Methods, Evidence, and Impact, Faculty of Health Sciences, McMaster University, Hamilton, ON L8S 4K1, Canada; 3 Department of Medicine, University of Health Sciences, Istanbul 34668, Turkey; 4 Institute of Health Policy, Management and Evaluation, Dalla Lana School of Public Health, University of Toronto, Toronto, ON M5T 3M6, Canada

**Keywords:** operations research, kidney transplantation, organ allocation

## Abstract

Background: Operations research techniques enable health care administrators to optimize resource allocation and to find solutions to staff and patient scheduling problems. We aimed to conduct the first systematic review of the international literature on the use of operations research for allocating deceased-donor kidneys. Methods: We searched the MEDLINE, EMBASE, and PubMed databases from inception to February 2023. Two reviewers independently screened the title/abstract and subsequently the full text of potentially eligible articles and abstracted the data. Quality assessment of the final set of studies was conducted using Subben’s checklist. Results: Of the 302 citations identified, 5 studies were included. These studies covered three themes, including (1) provider-facing decision aids to determine the timing of transplant for single or multiple patients; (2) system-level planning on kidney allocation based on blood type matching rules; and (3) patient-facilitated wait times estimation using incomplete information. Markov models, sequential stochastic assignment models, and queuing models were amongst the most used techniques. Although we found all included studies to meet Subben’s criteria, we believe the checklist in its current form lacks items to assess the validity of model inferences. As such, we ended this review with a set of practical recommendations. Conclusions: Our review demonstrated the utility of operations research techniques in assisting the system, healthcare providers, and patients in the transplantation process. More research is needed to reach a consensus on a model that can be used to support the decision-making of different stakeholders for efficient kidney allocation, with the ultimate goal of reducing the gap between kidney supply and demand and enhancing the population’s well-being.

## 1. Introduction

### 1.1. Kidney Transplantation and Allocation

Chronic kidney disease (CKD) is described as irreversible kidney damage caused by structural or functional abnormalities that persist for 3 months or longer [1]. At least 10% of the world population was affected by CKD in 2022, which implies a significant global burden of diseases, including mortality, morbidity, and exceedingly high healthcare resource use [2]. Using the estimated glomerular filtration rate (eGFR) in combination with other clinical indications, CKD is classified into five stages, with stage 5 disease (i.e., end-stage renal disease) typically requiring an immediate initiation of renal replacement therapies, including either dialysis or receipt of kidney transplantation, to sustain life [3]. Both living- and deceased-donor kidney transplantation are known to lead to superior life expectancy, but an efficient allocation of kidneys to suitable candidates is complex [4]. Due to the growing gap in the supply and demand for donor kidneys, several policy paths have been designed to overcome blood type and tissue incompatibility issues as a means to maximize the use of scarce kidneys [5,6,7,8,9,10,11,12,13]. For example, models of kidney exchange use a cyclic transplant procedure with cross-over transplantation to find compatible donor–recipient pairs [14,15]. This means a transplant candidate who is immunologically incompatible with their own donor’s (often a family member) kidney is given a kidney from another patient’s donor who would, in return, receive the kidney from the first patient’s donor. In general, transplanting patients who have been ultimately matched with a less compatible kidney requires more immunosuppressive agents, and a closer follow-up to ensure an adequate post-transplant outcome [16,17]. The burden of care and health risks can be substantial, especially for certain patient subpopulations that are known to be susceptible to surgical complications, transplant rejections, and poor patient and graft survival outcomes, such as older adults and those with diabetes, heart disease, and hypertension [18,19,20,21]. This means shared decision-making between providers and patients/caregivers is necessary to optimize management outcomes and patient satisfaction. In fact, details on the treatment effectiveness, safety, pill burden, adherence to potentially lifelong therapy, and cost issues with the current regimen need to be explained to patients [22].

For the health system, a fair and efficient kidney allocation and transplantation process requires close attention to both medical and nonmedical factors [4,23]. Specifically, the allocation of kidneys must follow an evidence-based clinical guideline that involves rigid blood- and tissue-matching principles; these medical factors are directly related to graft survival, which will eventually affect patient quality of life and longevity [3]. Nonmedical factors, including considerations on ethics, economics, and logistics, on the other hand, would drive kidney allocation decisions to affect equity and fairness in the long run [4,24]. As such, national kidney allocation policies must consider all relevant factors in formulating the best strategies while ensuring feasibility in practice [3]. Notably, the use of a geographically based selective system can improve allocation efficiency by prioritizing local patients, and then regional patients, and lastly, the entire national pool of waitlisted patients. A blood-type-based matching scheme is also widely implemented, which entails the prioritization of transplant candidates with identical blood type to their donor in addition to having zero human leukocyte antigen (HLA) mismatched; this is followed by patients with a compatible blood type and zero antigen mismatched, and finally, other blood type compatible candidates [3]. Due to these rules, transplant wait times are known to vary according to the blood type, with type B patients typically spending the longest time waiting on dialysis relative to other patients, especially those with type AB blood (B > O > A > AB) [25,26].

### 1.2. Operations Research

Operations research enables health care administrators to optimize resource allocation and finding solutions to staff and patient scheduling [27,28,29,30,31,32,33]. In the context of kidney transplantation, these goals broadly translate to improving the utilization of kidneys, a valuable and scarce healthcare resource, in a clinically gainful, cost-effective, and time-efficient manner [34]. Operations research can handle both soft and hard constraints in an optimization setting. Hard constraints accommodate little or zero flexibility, while soft constraints are subject to adjustments based on their weight and impact on the objective function. Therefore, when violated, soft constraints are penalized based on their importance on the outcome (or objective function) using preassigned weights based on various assumptions. In kidney allocation, both hard and soft constraints are relevant [34].

The objective function determines the objective of making decisions by specifying the relationship between decision variables. In the context of kidney transplantation, the objective function needs to reflect the goal of maximizing the number of successful kidney transplants without compromising principles of equity [3,34]. Additional objectives may include reducing unwarranted regional variability around wait times, improving access to kidney transplantation, and maximizing patient-important outcomes. To solve optimization, models are developed where parameters are populated by real-world data.

Other than optimization models, other operations research techniques may be valuable for kidney allocation. For example, queuing models in combination with simulations are ideal for sensitivity analysis and addressing variability.

### 1.3. Review Objectives

Our objectives were as follows: (1) to describe the current landscape in using operations research methods to tackle issues pertaining to deceased-donor kidney allocation; (2) to assess the reporting quality of the existing literature using an established appraisal tool; and (3) to identify if this appraisal tool can indeed be reliably applied to judge the quality of literature in kidney allocation, and if not, to provide suggestions on how to improve components of this tool.

## 2. Materials and Methods

### 2.1. Review Design and Literature Searches

This systematic review was performed according to the Preferred Reporting Items for Systematic reviews and Meta-Analysis (PRISMA) version 2020 guideline [35]. We searched for English-language, peer-reviewed journal articles on the MEDLINE, EMBASE, and PubMed databases from inception to 19 February 2023 (see database-specific search strategies and results in Appendix A). For every eligible study we identified from the database search, one reviewer (N.S.) further examined its reference list to retrieve more relevant studies. The same reviewer also performed an in-depth search on Google Scholar, Science Direct, and Web of Science.

### 2.2. Eligibility Criteria

We included original investigations that explicitly applied an operations research analytical pipeline and techniques to study the allocation of deceased-donor kidneys. Specifically, to qualify for inclusion, studies must have demonstrated how they solved a specific decision-making problem pertaining to kidney allocation, such as finding the best quality threshold level to accept or reject a donor kidney or minimizing transplant wait times through an improved managerial system. Furthermore, studies must have presented sufficient quantitative details, including but not limited to the mathematical model/algorithm, simulation process, and statistical analysis results. We also required studies to formulate clear real-world implications. Case reports, quality improvement studies, conference abstracts, and non-English articles were excluded. We also excluded studies that only presented a graph theoretic-based tool without further elaborations, those conducted on living-donor kidney transplants or exclusively on pediatric patients (age < 18) as the recipients of transplantation, and studies that assessed solid organ transplantation in general without conducting separate investigations and providing the relevant results on deceased-donor kidney transplantation.

### 2.3. Study Selection and Data Synthesis

One reviewer (N.S.) performed the literature search and imported all citations into the EndNote software, where duplicates were removed automatically. The same reviewer then transferred the data to the Covidence platform, where two reviewers (N.S. and R.F.) independently screened the title and the abstract and then proceeded with full-text screening. Disagreements were resolved through discussion between the two reviewers. A qualitative narrative approach was employed to summarize the included studies. Since we anticipated a relatively small set of studies to be ultimately included in this review, we briefly summarized the rationale, method, findings, implications, and limitations of each study in our evidence synthesis. Furthermore, we grouped studies based on their primary objectives with respect to the target user (patients, health care providers, or system planners/decision-makers) and the type of issue their tools were designed to tackle.

### 2.4. Risk-of-Bias Assessment

One reviewer (N.S.) employed Subben’s checklist, which had been specifically designed for appraising the methodological quality of operations research in a risk-of-bias assessment [36]. This checklist seeks to comprehensively assess the reporting adequacy of the following domains: Relevance, Background, Motivation and Remedy, Hypothesis, Methodology, Realization, and Analysis. The Relevance domain should present the objective of the present study in relation to the potential benefit of the study results to address an identified need in the real world. The Background domain provides supporting and historical information. Motivation and Remedy should elaborate on the gaps in knowledge and/or weaknesses of the current methodology in addition to proposing appropriate remedies. The Hypothesis domain should contain a clear definition of the research question. The Methodology domain should provide all relevant analytical details. The Realization domain demonstrates the new findings and the implications. Finally, the Analysis domain should cover the validation of results, conclusions, and future directions. Beyond assessing the quality of our included studies, we also attempted to identify the limitations of the checklist per se in the context of operations research applied to kidney allocation.

## 3. Results

### 3.1. Search Results

**Figure 1:**
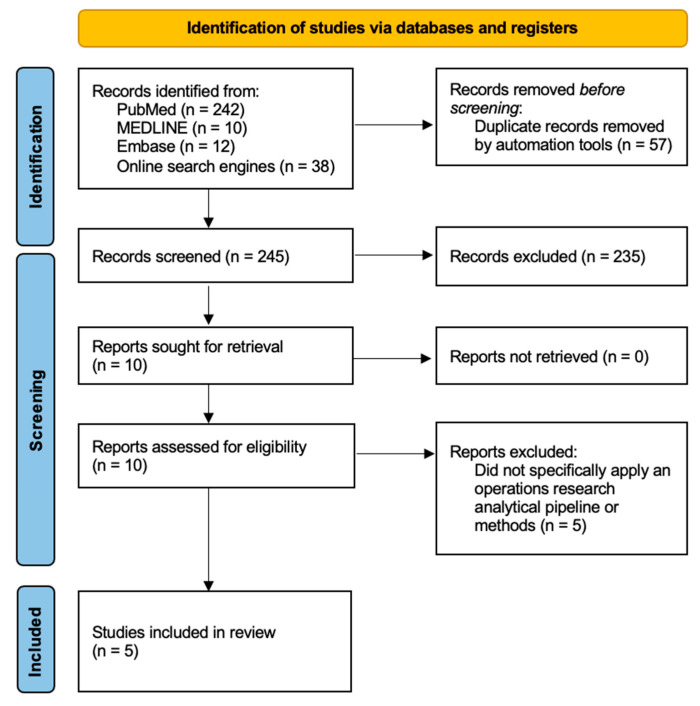
A PRISMA diagram documenting the selection of studies in this review.

The database searches yielded 264 citations, of which 245 were unique (Figure 1). Searching on various online engines revealed another 38 papers. Through title and abstract screening, we identified 10 potentially relevant studies whose full text was subsequently examined. 5 studies were excluded due to not specifically using an operations research analytical pipeline or methods to conduct their investigations. As such, five studies were retained with years of publication ranging from 1996 to 2019.

### 3.2. Summary of Findings

Of the five studies we included, three were conducted in the US [22,37,38] and one each in Canada [39] and Israel [40]. The three US studies have developed their models in the context of the United Network for Organ Sharing (UNOS) system; furthermore, all of them have drawn administrative data to provide illustrative examples as a way to elaborate the real-world implications of their models in a uniquely US setting. The two non-US studies did not mention any particular jurisdiction or health system that their models were based upon; rather, they demonstrated their model in a hypothetical organ allocation system.

The five studies employed operations research methods to explore three themes pertaining to optimizing a fair distribution of deceased-donor kidneys.

#### 3.2.1. Provider-Facing Decision Aids to Determine the Timing of Transplantation for Single or Multiple Patients

For individuals who are deemed to be suitable transplant recipients, they are confronted with a decision on when to accept a kidney. Operations research can aid this individualized decision-making process by providing insights on when to perform the transplantation. Ahn and Hornberger [22] developed a semi-Markov model with five states (alive on dialysis and waitlisted for a transplant, not eligible for a transplant and on dialysis as destination therapy, received a functioning transplant, transplant failed, and death) to examine a threshold level of accepting (or rejecting) a kidney based on a patient-specific quality-adjusted life-years (QALY) index. This model is able to accommodate specific characteristics of patients, including age and pre-existing comorbidity, as those who are younger and with less comorbid conditions are known to have more favorable transplant outcomes (including shorter length of hospital stay and better survival). Additionally, this model can adjust the output according to the risk-aversion profile of individual patients since some of them may be willing to accept an offer for transplantation knowing the kidney used is of less desirable condition. Because in most of the current allocation policies the transplant care team is ultimately responsible for making the transplant timing decisions, this model is potentially useful as a provider-facing decision aid.

Later, a sequential stochastic assignment model was developed by Su and Zenios in 2005 [37]. This model enhanced the one created by Ahn and Hornberger by simultaneously considering multiple transplant candidates in the decision-making process. A choice-based system was used to mathematically penalize patients who rejected an offer. Furthermore, those who are willing to wait longer may receive a better kidney if they live long enough to get a second offer. This added flexibility of this model may improve its practical utility when compared to the one previously developed by Ahn and Hornberger.

#### 3.2.2. System-Level Planning on Kidney Allocation Based on Blood Type Matching Rules

Stanford et al. [39] described a queuing model as a managerial planning tool for stochastically arriving kidneys from deceased donors. This system was based on blood type matching, meaning that it would allow a set of compatible pairs and reject incompatible pairs. For example, a transfer between blood types O and B or A and AB is permitted, while a first-come, first-served queuing rule is enforced for a donor kidney with multiple blood type-matched candidates. The G/M/1 rule was employed to ensure the stability of queues in the transplant system; specifically, the consecutive time for the availability of the same kidney (i.e., sojourn time) was assumed to follow an exponential distribution. The major weakness of this model was the assumption of constant queuing time between types B and O that was assumed to follow a single Poisson distribution; this may not hold true in the real world.

A more sophisticated analysis can be found in Perlman et al. [40], who assumed two Poisson processes: one was for patients (kidney candidates), and the other was for blood type (kidney resources). Matching according to the HLA was also explicitly considered in the analytical process requiring the delivery of the kidney to the best HLA-matched candidate. However, this paper only considered types B and O, rendering the model incomprehensive as it ignored other blood types that would require permissive cross-transplantation to improve equity.

#### 3.2.3. Patient-Facilitated Wait Times Estimation Using Incomplete Information

An efficient transplantation allocation system must tackle the inevitable gap between the exceedingly high demand for deceased-donor kidneys and the limited supply. Therefore, wait times estimation is very important for the system and for patients wishing or are currently enlisted for a transplant. In the United States and many other jurisdictions kidneys are first allocated based on blood type compatibility, but additional factors, such as the location of this patient, their willingness to accept a marginal kidney, and the relative urgency of this patient when compared to other competing candidates, also affect the wait times.

Bandi et al. [38] used the multiclass multiserver queuing theory under a transient regimen and a mixed-integer programming formulation to help transplant candidates to estimate their waiting time. Assuming the waitlisted patients have access to some, but not all, system information (such as their current ranking on the waitlist), the authors used historical data to build and validate a model under a first-come, first-served allocation rule. This model requires users (i.e., transplant candidates) to enter one or more of the following data: their own kidney quality preferences, blood type, location, and rank on the waitlist. Then, the model would estimate patients’ wait times based on these inputs.

### 3.3. Quality Assessment

We employed Subben’s checklist and found all domains were adequately addressed in each study. Nevertheless, we believe the checklist in its current form may be inadequate to comprehensively judge the quality of the study and rigor in methodology. Specifically, the checklist has only one domain on checking the appropriateness of research methodology. Due to the complexity of operations research studies and the fact that extensive mathematical considerations and assumptions are often required to underpin the statistical model, we believe that subtypes of the main checklist can be created to tailor to various designs and analytical components (queuing theory, linear programming, simulation model, and etc.) Furthermore, the current checklist contains a single Analysis domain that aims to capture information about the validation of results, conclusions, consequences, and applications in the assessed study. We believe that, to provide more in-depth appraisal results, multiple domains should be created so information on the conclusions, consequences, and applications can be evaluated separately. We also explored the usability and face validity of this tool and found both aspects were satisfactory. However, the tool can be improved by avoiding multitasking under one domain and providing more information about the assessment process for each domain.

## 4. Discussion

To the best of our knowledge, the current study represents the first systematic review of applications of operations research of deceased-donor kidney transplantation. Beyond providing an evidence synthesis, we explored the gaps in the literature and assessed the quality of the published papers. Moreover, we critically appraised the completeness of Subben’s checklist specifically with respect to the five studies we reviewed and in the field of kidney transplantation. Our appraisal results revealed the limitations of the checklist that warrant further consideration.

We excluded studies that used graph theory to solve kidney exchange problems. This is because the data-drive graph theory is static, and therefore, is not able to address all relevant issues in the dynamic kidney exchange process. As such, all empirical studies considered in this review were model-driven analyses providing a dynamic solution to kidney allocation.

We identified five published studies that employed operations research techniques within the scope of this review. Two studies developed a provider-facing decision aid to determine the timing of transplantation. Specifically, an optimal quality threshold for kidneys is identified for a single patient or for multiple patients simultaneously to help providers formulate the most suitable transplant plan. Another two studies developed a blood type-based queuing model to support system-level allocation planning for stochastically arriving kidneys from deceased donors. The model also has the capacity to accommodate cross-transplantation between type B and O and permissive cross-transplantation to improve equity. Finally, we found one paper that focused on a patient-facilitated wait times estimator based on the multiclass multiserver queuing theory with a mixed-integer programming formulation.

Four of the five studies were based in North America, and only three of these studies have demonstrated real-world applications of their models using administrative data. In other words, current applications of operations research in deceased-donor kidney transplantation remains highly theoretical and experimental, despite the potentially substantial utility of operations research in a real-world implementation. Furthermore, since we did not find any studies conducted in an exclusive setting of developing countries, this remains a significant limitation in the literature since chronic kidney disease is known to be especially burdensome for these jurisdictions that also tend to lack a well-established and rigorously managed organ allocation system [2].

### 4.1. Study Implications

Operations research methods have the potential to improve the allocation of deceased-donor kidneys for patients, health care providers, and the system. These techniques are versatile, and as such, can accommodate a variety of nuanced statistical inputs, including the natural history of chronic kidney disease, stochastically arriving donor kidneys, quality of kidneys, levels of system oversight, expert judgments, and the preferences of patients. However, according to our review, the real-world usability of these tools is largely unclear, especially for non-US health jurisdictions. Even for the US, the current evidence is very scarce, and the data used to illustrate these models (if any) are dated. Since kidney allocation is a highly complex, multilevel process, implementation of an innovative operations research-based solution warrants extensive evaluations on its efficacy, feasibility, acceptance by health care providers, cost-effectiveness to society, and the potential unintended consequences to the system and patients. Particularly, researchers and decision-makers must carefully examine the equity of kidney allocation solutions generated by these tools to completely rule out the possibility of them favoring a certain group of patients over others. This requires an upfront appraisal of such tools before their deployment into the real world, as well as regular and frequent inspections during their implementation to ensure consistently satisfactory performance. These procedures are beyond the scope of this systematic review, but researchers may want to consider quality improvement studies, field experiments, feasibility trials, health technology assessments (including cost–utility analyses and budget impact analyses), and qualitative interview studies with clinical leaders, providers, and patients to conduct the appraisal.

Also for researchers, the next step in this line of research may be to establish operations research as one of the standard methods for solving specific managerial problems in kidney allocation. An explicit and rigorous methodology is needed for making valid conclusions and obtaining reproducible results. At this juncture, it is unclear how to assess the quality of evidence within the scope of operations research. Therefore, a well-developed and validated tool is needed. This will eventually affect health policies for solid organ transplantation at the local, regional, and global levels.

### 4.2. Recommendations

We highlighted the weaknesses of Subben’s checklist as it is applied to evaluating the reporting quality of operations research studies in kidney allocation. We believe the checklist in this current form lacks items to assess the validity of statistical inferences drawn from the modeling results. Furthermore, we believe this checklist needs to be tailored based on the methods used in the empirical analysis. To do so, different subtypes of the main list may be developed and validated for various operations research techniques.

We provided the following six recommendations for strengthening the application of operations research in finding solutions for kidney transplant allocation:

1. Prioritize the use of operations research to answer managerial questions related to kidney transplantation.
2. Test the usability of Subben’s checklist in different medical contexts.
3. Revise Subben’s checklist by giving more weight to domains that judge the rigor in methodology, especially on the validity of inferences.
4. Enhance Subben’s checklist by giving clear instructions on how to assess the nuances in the study methodology.
5. Conduct a more comprehensive sensitivity analysis to quantify model uncertainties (e.g., by varying the level of confidence in the model assumptions and changing the underlying distribution of model parameters).
6. Establish the real-world reliability of model-driven dynamic solutions derived from operations research.

### 4.3. Strengths and Limitations

We would like to include the strengths of our study. This review is the first comprehensive and structured review of applications of operations research techniques in deceased-donor kidney allocation. The robustness of our evidence synthesis results is enhanced by our systematic search strategy, explicit study eligibility criteria, inclusion of two reviewers to conduct screening and extraction in duplicates, and the recent search date (February 2023) to comprehensively capture all relevant studies. We believe our synthesis results are novel in the review literature and as such, have unique implications for policy, practice, and research that we have also outlined.

There are several limitations of our study. First, we presented a clear focus on kidney transplantation and thereby ignored the issues with the upstream and downstream departments. For example, once a patient has received a transplant, multiple healthcare departments, including but not limited to the surgical team, intensive care unit, primary care doctors, dieticians, and home care team, are more or less involved. Therefore, future review studies may want to expand our review by including operations research in all relevant renal care units and encompassing the entire trajectory of the kidney transplantation process. Furthermore, this review did not consider other types of kidney transplantation, such as living-donor transplantation, multi-organ transplantation (such as a kidney–pancreas transplantation), and transplantation via a nontypical pathway (such as using the kidney from an expanded criteria donor or through a kidney-paired exchange program). Due to the nuances of these transplantations, we believe rather than slightly modifying the existing models for deceased-donor kidney transplantation, a separate analysis should be conducted to devise completely new systems to be used in these scenarios. Additionally, we employed a designated tool for quality assessment (i.e., Subben’s checklist). However, this tool is not validated specifically for the context of kidney transplantation. As a result, we critically assessed the completeness of this tool and gave suggestions on how to potentially enhance its components. Next, we do recognize that engineering libraries such as IEEE Xplore host some managerial literature that is highly technical [41]. Since we did not specifically search for these databases, future review studies may want to mitigate this limitation of ours. Finally, despite the recent literature search date, our review did not reveal any studies that involved the COVID-19 pandemic. Notably, none of the five studies have simulated a pandemic (or a similar public health emergency or catastrophic social event) scenario or explicitly incorporated a pandemic-related parameter in their mathematical models. Future operations research related to organ transplantation needs to account for the pandemic-related interruptions in the procurement, allocation, and donation of organs for transplantation to provide results that are more meaningful in the post-pandemic contemporary era.

## 5. Conclusions

The present study is the first to systematically review operations research studies on deceased-donor kidney allocation. Results of this review suggest that the limited number of existing studies are of relatively high quality, although the appraisal tool (Subben’s checklist) itself may be insufficient in assessing the reporting quality of empirical analyses in this particular area. We gave concrete recommendations for operations research investigators, evidence synthesis experts, and decision-makers, including the potential to expand Subben’s checklist to tailor to different study designs. More research is needed to reach a consensus on how to adopt an operations research lens to tackle specific issues in kidney allocation.

## Data Availability

Not applicable.

## Appendix A. Literature Search Strategies

Database: PubMed <1966 to 19 February 2023>

Search Strategy:

--------------------------------------------------------------------------------

(operations research) AND (kidney transplantation) 242

***************************

Database: Ovid MEDLINE(R) <1996 to 19 February 2023>

Search Strategy:

--------------------------------------------------------------------------------

1 operations research.af. (5161)

2 kidney transplantation.af. (113181)

3 1 and 2 (10)

***************************

Database: Embase <1974 to 19 February 2023>

Search Strategy:

--------------------------------------------------------------------------------

1 operations research.af. (4172)

2 kidney transplantation.af. (154114)

3 1 and 2 (12)
